# Supplementary material for: Outpatient antibiotic prescribing during the first two years of the COVID-19 pandemic: A nationwide register-based time series analysis
Source: PLoS One. 2023 Dec 18;18(12):e0296048. doi: 10.1371/journal.pone.0296048 (PMC10727428; doi:10.1371/journal.pone.0296048)
Supplement: Attachment — Submitted filename: Response to reviewers.docx [file pone.0296048.s001.docx]

PONE-D-23-26461

Outpatient antibiotic prescribing during the first two years of the COVID-19 pandemic: A nationwide register-based time series analysis

PLOS ONE

**Response to Reviewers**

The authors would like to thank the reviewers for their time and the comments that have helped us to improve our manuscript titled “Outpatient antibiotic prescribing during the first two years of the COVID-19 pandemic: A nationwide register-based time series analysis” (Manuscript Number: PONE-D-23-26461). We have considered all the reviewers’ comments and made changes in the revised manuscript accordingly. We believe these changes address the questions raised by the reviewers. Please see our detailed responses to the comments of each reviewer below.

| Reviewer #1 | | |
| --- | --- | --- |
|  | Comment | Answer |
| 1 | Abstract  Authors state “ Future studies should 16 explore the possible effects of the decline on antimicrobial resistance.” I am wondering of this is more important than understanding reasons for decline here. Steps like a step further than what links more closely with study aim. | We thank the reviewer for the comment. We have now revised the abstract conclusions. Please see the last sentence of the abstract in page 2. |
| 2 | Introduction  The authors mention increase and decrease in antibiotic prescription. It would be useful if they discuss what are reasons for such contradicting outcomes in different countries? | We have added discussion on possible reasons of varying antibiotic prescribing practices in different countries to the introduction. Please refer to page 3, lines 27-29 (Revised manuscript with track changes). |
| 3 | Method  The paper has used robust methods for assessment of antibiotic prescribing across different time periods.  Does the study population include all population in Finland that is eligible… based on criteria specified in the manuscript. Or there was some sampling performed. May be useful to clear this. | We thank the reviewer for pointing out, that this needs clarification. The study population includes all people in Finland to whom antibiotic prescriptions had been prescribed for outpatient care during the study period. We have now clarified this also to text (page 5, lines 71-73). |
| 4 | Results  Presented well | Thank you for this comment |
| 5 | Discussion  Again little discussion on explaining results. | We have now updated the discussion and added some more discussion on explaining the results. (Page 11, lines 193-194 and 208-210) |
| Reviewer #2 | | |
|  | Comment | Answer |
| 1 | In the Introduction section it is presented only azithromycin as a product with an increased consumption.  Question 1. Has authors reviewed consumption of quinolones (Cipro, Levo, Moxi – floxacins) and carbapenems (Imipenem) for an increased consumption as well? | Thank you for this question regarding possible increased consumption of quinolones or carbapenems.  We reviewed literature for outpatient care consumption of quinolone antibiotics and did not find evidence of increased consumption of quinolones during COVID-19 pandemic.  Carbapenems are not prescribed or used in outpatient care in Finland. We did not have access to hospital data, and for that reason carbapenem antibiotics are not included to this study. |
| 2 | In the lines 45 – 48 are mentioned measures for the pandemics outbreak.  Question 2. Were there also measures (Such as guidelines, orders etc) for antibiotic prescription for or against covid-19 in Finland? | In Finland, there has not been problems with large-scale irrational prescribing of antibiotics. Antibiotics are not effective against viruses like the one that causes COVID-19, but there have been guidelines for treating COVID-19-related secondary bacterial infections in hospital settings. For example cefuroxime was recommended as the first-line antibiotic treatment for hospitalized patients with secondary bacterial pneumonia. Please see page 4, lines 59–64. |
| 3 | Data, Methods, statistical analysis are presented very well | Thank you for this comment. |
| 4 | Discussion to be linked more with conclusion – or conclusion to be expanded more according to the findings. | We have now updated conclusion so that it is more according to the findings. |
